# Diversity Analysis of Streptomycetes and Associated Phosphotranspherase Genes in Soil

**DOI:** 10.1371/journal.pone.0035756

**Published:** 2012-04-23

**Authors:** Paris Laskaris, Takuya Sekine, Elizabeth M. H. Wellington

**Affiliations:** School of Life Sciences, University of Warwick, Coventry, United Kingdom; Cinvestav, Mexico

## Abstract

An attempt was made to verify the observation that *Streptomyces griseus* was prevalent in soil based on isolation work. A genus-specific PCR was developed for *Streptomyces* based on the housekeeping gene *atpD* and used to investigate species diversity within selected soils. The presence of *S. griseus* was investigated to determine coexistence of resistance-only streptomycin phosphotransferase (*strA*) in the same soil as streptomycin producers. Two additional PCR-based assays were developed; one specific for *strA* in association with production, the other for more diverse *strA* and other related phosphotranferases. Both the *S. griseus atpD* and *strA* genes were below the PCR detection limit in all soils examined. A number of more diverse phosphotransferase genes were amplified, a minority of which may be associated with streptomycin production. We conclude that neither streptomycin producers nor *S. griseus* are prevalent in the fresh or chitin and starch-amended soils examined (less than 0.1% of soil actinobacteria). One of the soil sites had received plantomycin (active ingredient: streptomycin) and diversity studies suggested that this altered the streptomycete populations present in the soil.

## Introduction

The *Streptomyces* genus is one of the most diverse taxa within the archaea and bacteria and currently contains approximately 591 species [1]. Streptomycete taxonomy had suffered from overspeciation, as producers of novel antibiotic compounds were described as new species and patented as part of the antibiotic discovery process [2]. Phenotypic and chemotaxonomic characters were used to classify streptomycetes, however different characters gave rise to different taxa [3] and the molecular approaches became established with DNA-DNA hybridization used for valid species description [4]. For large scale studies sequences used included the 16 S rRNA gene [5], [6], [7], [8] the 16 S–23 S intergenic spacer region [9], [10], [11] random amplified polymorphic DNA [12] and protein-coding genes such as *rpoB* (RNA polymerase β subunit) [13], [14] or *trpB* (tryptophan synthase β chain) [15]. These methods have now been superseded by multi-locus sequence analysis (MLSA) [16], [17], [18], as the use of multiple gene sequences helps to buffer distortions on phylogeny generated by recombination [19].

The study of in-situ diversity of soil streptomycetes has not previously been undertaken, although streptomycetes in situ were reported as part of 16 S rRNA-based total microbial community studies [20], [21], [22], [23], [24], [25]. Within genera, in situ diversity is problematic due to the conservation of the 16 S rRNA gene [4]. Typing methods, such as MLSA, requiring multiple sequences per strain are not feasible; therefore our aim was to find an alternative gene target to compare diversity in situ in soil with that previously described by strain cultivation methods. The *atpD* gene, encoding the ATP synthase beta chain, was used in a previous study for MLSA that demonstrated its sequence divergence was sufficient to provide higher phylogenetic resolution than the 16 S rRNA and allow the generation of streptomycete-specific primers [17].

A key feature of the streptomycetes is the high diversity of resistance genes found within the genus [26]. This resistome is undoubtedly associated with prolific antibiotic production [27]. The first actinobacterial antibiotic isolated was from a streptomycete, *Streptomyces griseus* [28]. A number of *S. griseus* strains possess the *strA* streptomycin resistance gene, a 6′ aminoglycoside phosphotransferase (APH(6′)), within the streptomycin biosynthetic gene cluster to avoid suicide [17]. The *strA* genes have coevolved in resistance-only and streptomycin-producing strains and *strA* appeared to be taxon-specific, with one exception in *Streptomyces platensis* strain CR50 which was attributed to horizontal gene transfer [17]. Streptomycin is used in horticulture to control *Erwinia amylovora* which causes fire blight in apple and pear trees and used to treat other bacterial infections [29]. The use of streptomycin in agriculture was responsible for driving increased prevalence of - mobile elements containing *strA*-*strB*, a 3′ and 6′ aminoglycoside phosphotransferase respectively, in phylloplane bacteria [30]. Another study found that the application of plantomycin, a mixture of streptomycin sulfate and tetracycline hydrochloride, in an orchard made no significant difference in the number of resistant streptomycete soil isolates, though streptomycin producers comprised a larger proportion of resistant streptomycete isolates in soils not amended with streptomycin [31]. We therefore hypothesized that the application of streptomycin would lead to a decrease in the ratio of streptomycin producers to resistance-only streptomycetes, as the latter would be selected for due to the larger quantities of streptomycin in soil. The prevalence of *strA* in the environment has not been examined. To address this, we extracted total community DNA (TCDNA) from a number of soils from which streptomycetes with *strA* genes were previously isolated [31] or that contained large numbers of actinobacteria [32]. We hypothesized that, since approximately 1% of randomly screened soil actinobacteria can synthesize streptomycin [33], both the *strA* gene and the streptomycin producers would be found across a variety of different soils. This study was carried out to determine whether there was an enhanced prevalence of non-producers to producers in soil to gain a wider perspective on the range of hosts carrying the *strA* gene. Selective isolation always carries some bias for the estimation of species distribution. High levels of streptomycin predominantly selected for producers, as non-producers were less resistant [17]. Direct molecular detection and diversity analysis allow a non-biased estimate of *S. griseus* distribution and *strA* diversity.

## Materials and Methods

### Site descriptions (Table 1)

**Table 1:** Soil sites screened for the *strA* and *atpD* genes [31], [32].

Location	Soil information	Abbreviation	Microcosm
Skopelos, Greece	Agricultural site	116	Yes
	Pine forest	Skop1	No
Cayo Blanco, Cuba	Scrubland	403	Yes
	Fir forest	415	Yes
Basilicata, Italy	Conventional agricultural site	602	No
El Aguilucho, Spain	Terracing plus pines with mycorrhiza	728	No
Santomera, Spain	Devegetated forest	767	Yes
	Forest	770	Yes
	Forest	773	Yes
	Bare land	774	Yes
	Scrubland	777	Yes
Dossenheim, Germany	Apple orchard where plantomycin was regularly applied	AR	Yes
	Control site with no plantomycin application	CR	Yes
Sourhope, Scotland	Limed soil	5A	No
	Control site with no liming	5B	No
Cotswolds, England	Well drained alkaline soil	C	No
Cryfield, England	Grassland	W	Yes

The AR samples were taken from an experimental agricultural site in Dossenheim, Germany, where plantomycin (Neudorff Pharmaceuticals), containing streptomycin sulphate and tetracycline hydrochloride had been used for two years before sampling to control fireblight caused by the apple pathogen *Erwinia amylovora*. The solution contained 212 g streptomycin sulfate kg^−1^ and was applied at a rate of 1200 m^3^ ha^−1^ (0.06% solution) annually [31]. The CR samples consisted of the same soil taken in Germany without antibiotic application. The CW soil samples were taken from a chalky loam agricultural soil with a pH of 8.0, and the C soil from grassland, both originating from Cotswolds, England [34]. The 5A and 5B samples consisted of a sandy silt loam collected from an experimental field in Sourhope, Scotland [35]. The 602 soil was sampled from agricultural soil in Basilicata, Italy cropped with *Triticum durum* for four years where conventional fertilizer was used [32]. The 767, 770, 773, 774 and 777 samples originated from an experimental field area is Santomera, (38°2′N, 1°12′W, 130 m elevation) in south-east Murcia region, Spain containing a relatively bare area (5% coverage), a shrub growing area (25%), as well as a pine forest (*Pinus halepensis* Mill, plant coverage ca. 70%) whose soil had a silt loam texture [32]. The 403 and 415 were sandy soils originating from Cayo Blanco, Cuba [17]. The soils selected for this study (Table 1) either contained large amounts of actinobacteria (Skopelos, Cayo Blanco, Bacilicata, Santomeras, Sourhope) [32] or had streptomycetes possessing *strA* previously isolated from them (Cayo Blanco, Dossenheim, Cryfield) [31]. All necessary permits were obtained for the described field studies. The UK Department for Environment, Food and Rural Affairs provided a licence to import, move and keep prohibited soil (other than for chemical and physical analysis). Licence No. PHL 208/6372 (09/2010).

### Preparation of soil microcosms

The soil water content was estimated by weight before and after water loss. 10 g of soil incubated at 105°C oven for 16 h, then cooled in a Dry-Seal Desiccator (Jenkons; Leighton Buzzard, UK) for 1 h. 10 g of non-dried soil was placed in a 50 ml Greiner centrifuge tube (Sigma-Aldrich) and enough water was added to bring the soil water content to 16%. 1% of the soil's weight in chitin (powdered α chitin from crab shells) and 1% in soluble starch (Analar) were added to select for streptomycetes. The tube was shaken until soil clumps broke up and the water, starch and chitin were well mixed. The tubes were placed in a 30°C incubator with their lids loose to allow the exchange of air and left to grow for 7 days in humid environment.

Microcosms were made with selected soils (Table 1) in order to select for streptomycetes and thus increase the probability of detecting *strA*. The AR and CR were sampled twice, to obtain higher resolution, the Santomera soils were sampled five times, once per site (767, 770, 773, 774, 777), the Cayo Blanco soils twice, again once per site (403, 415), and Skopelos (116) and Cryfield (W) were sampled once as soil was taken from a single site for them.

To prepare seeded soil microcosms, 10 g of soil was autoclaved twice and an *S. griseus* DSM 40236 spore suspension added to bring the soil water content to 16% and 10^7^ spores g^−1^. The seeded universal bottles were placed in a 30°C incubator with their lids loose to allow the exchange of air and left to grow for 7 days in humid environment.

### DNA extraction from soil

DNA was extracted from soil using the UltraClean Soil DNA Isolation Kit (MO-BIO) as stated in the manufacturer's instructions. To check the quality and amount of DNA, 5 µl of the eluate was electrophoresed. The DNA concentration was determined using a Nanodrop spectrophotometer (ND-1000, Nanodrop Technologies) and samples stored at −20°C.

### Detection of *strA* and *atpD*

The *strA* and *atpD* PCR products from all microcosm soil DNA extracts were cloned and a random selection sequenced. The AR and CR soils were examined to determine whether the use of plantamycin at that site had selected for resistance. The blastn algorithm was used to identify the closest homologues to all the *strA* and *atpD* sequences on GenBank and these were included in the phylogenetic trees.

Reaction mixes were made with 25 µl PCR Master Mix (Promega, Madison, WI, USA), 2.5 µl DMSO, 2 µl of BSA (bovine serum albumin) and 100 pM of each primer (Table 2) in 50 µl total volume. The cycling protocol used was the same for all primers with only the annealing temperature varying (Table 2): 10 min denaturing step followed by 35 cycles of 60 s at 95°C, 45 s at TA and 90 s at 72°C followed by a final extension step for 10 min at 72°C. The PCR products were run on a 1% agarose gel and the product bands were cut out and extracted using the QIAquick Gel Extraction Kit (QIAGEN; Venlo, Netherlands) as per manufacturer's instructions. The product was dialyzed for 15 min using 0.025 µm VSWP nitrocellulose membranes (Millipore; Billerica, MA, USA) placed on sterile water. PCR products were cloned using the PGEM T-easy vector system and plasmid DNA extracted using the QIAGEN Mini-prep kit as per the manufacturer's instructions to isolate single amplicons for sequencing. Sequencing was performed on 5 or 6 clones from each site, with approximately 30 sequences obtained from AR and CR to enable comparison of the effects of plantomycin addition. Sequencing utilized 50 ng of PCR product, 5.5 pM of primer and the BigDye Terminator v3.1 Cycle Sequencing Kit (Applied Biosystems; Foster City, CA, USA) on an ABI PRISM 3130xl Genetic Analyzer as stated in the manufacturer's instructions. Both the SP6 and T7 primers were used for sequencing to ensure there were no sequencing errors. The sequences were submitted to GenBank with accession numbers JN695275 to JN695392 for the *atpD* sequences and JN695393 to JN695497 for the *strA* sequences.

**Table 2:** Primers used to screen DNA extracted from soils.

Gene	Primer	Sequence	Annealing	Size	Source
*strA*	strA_F	GCG GCT GCT CGA CCA CGA C	63°C	570	[31]
	strA_R	CCG TCC TCG ATG TCC CAC AGG G			
*strA*	strA_F2	AGG CCT CCC TCG TGS TGC	60°C	615	[53]
	strA_R3	SGT CAG CAG GTC GAA GCG			
*atpD*	atpD_F	AAG ACC GAG ATG TTC GAG AC	56°C	466	[17]
	atpD_R	CCA TCT CGT CGG CCA GGT TC			

## Results

### Screening of soils for streptomycete diversity using *atpD*

**Figure 1:**
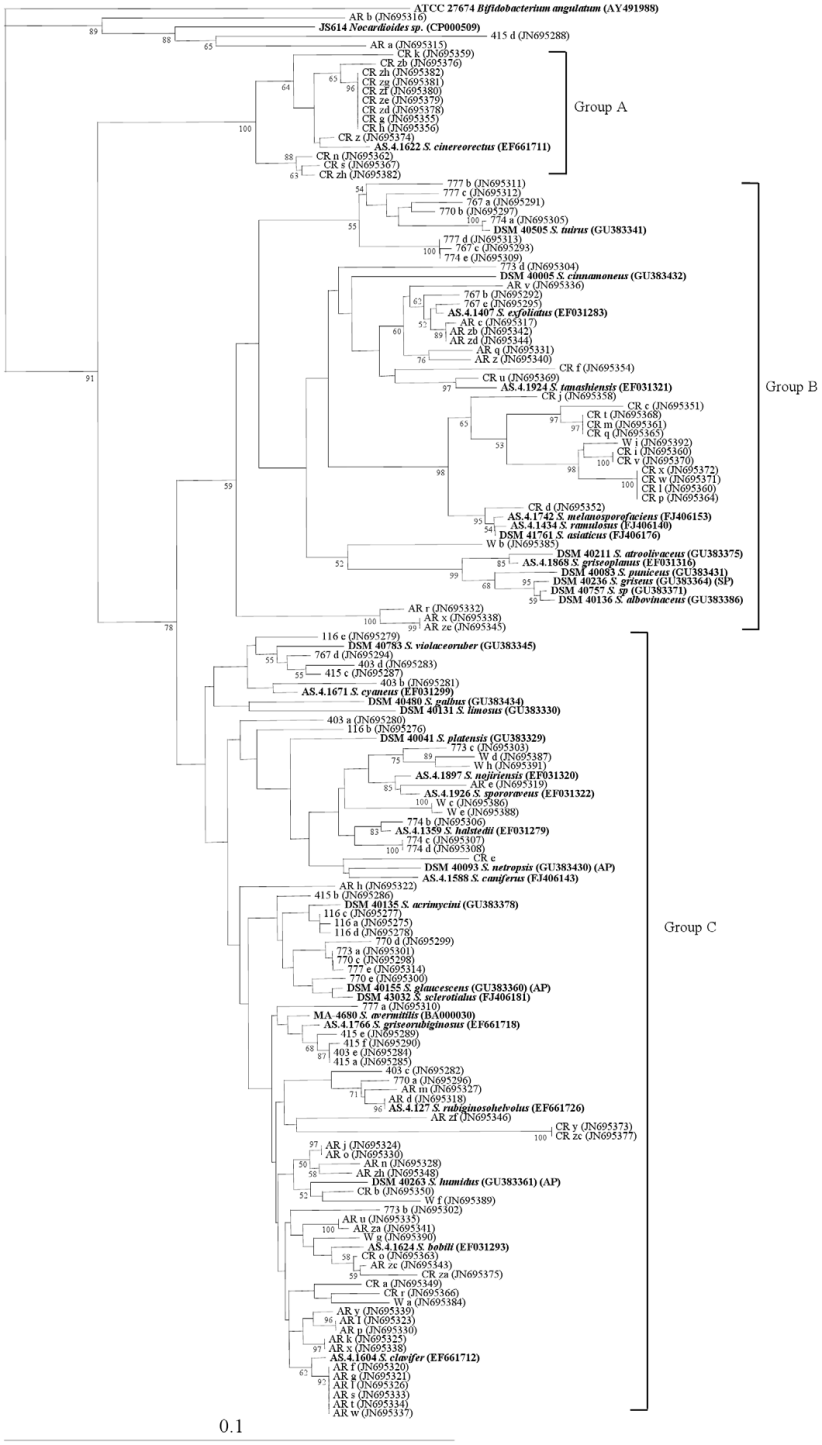
Diversity of cloned *atpD* sequences from all soil microcosms with markers from GenBank in bold. (SP) = Confirmed streptomycin producer, (AP) = Confirmed producer of other aminoglycoside [51], [52]. Accession numbers in parentheses. The tree was constructed using the neighbour-joining method; the numbers besides the branches indicate the percentage bootstrap value of 10000 replicates. The scale bar indicates 10% nucleotide dissimilarity.

The *atpD* gene was successfully amplified from all the soils and enriched microcosms examined, indicating that streptomycetes were present at detectable levels. The *atpD* primers demonstrated high specificity to the *Streptomyces* genus. Only 3 out of 107 (2.8%) sequenced amplicons fell outside it; these belonged to the actinobacterial genus *Nocardioides*. After excluding the *Nocardioides* sequences, the amplicons were divided into three major groups (Group A, B, C) according to the nucleotide tree (Figure 1).

**Figure 2:**
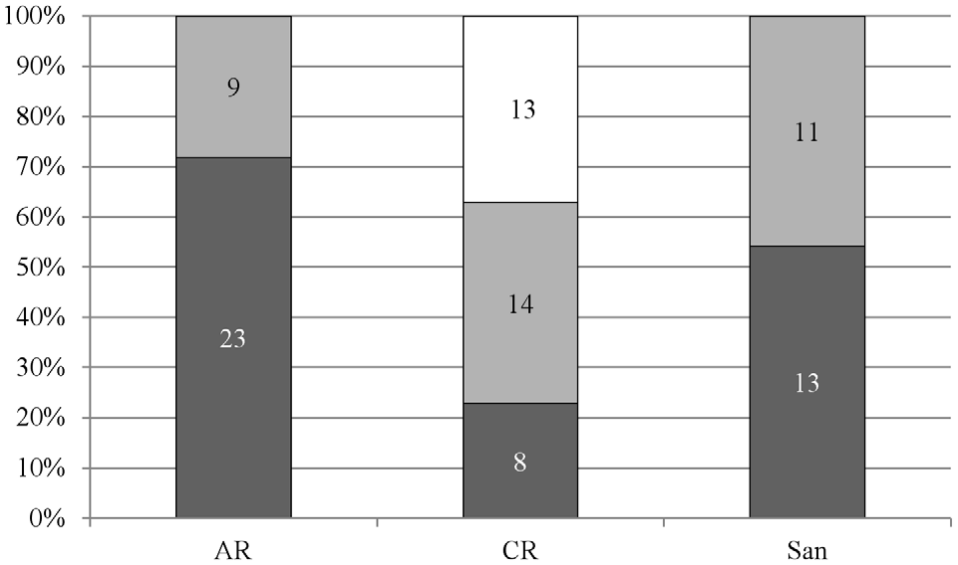
Distribution of the *atpD* sequences isolated from each soil site on the 3 main groups identified on the nucleotide phylogenetic tree (**Figure 1**). ‘San’ indicates all the Santomera strains (767, 770, 773, 774, 777). Group A in white, B in light grey and C in dark grey. The *Nocardioides* sequences are not included.

The *atpD* sequences from the same area clustered together, especially in Group A and B, though not in C (Figure 1). There were significant differences in the frequencies of *atpD* sequences between the AR and CR sample sites for Groups A and C, suggesting that plantomycin application may have affected the composition of the streptomycete populations in terms of diversity. There were also significant differences in the number of strains forming part of Group A, B and C between different areas but not between different sample sites within a given area (with the exception of AR and CR soils) (Figure 2, Table 3). This demonstrates that the population composition of streptomycetes varies between different regions. None of the sequenced amplicons fell within the same clade as *S. griseus*. The remaining *atpD* sequences either clustered with other species, such as *S. violaceoruber*, or failed to cluster with known sequences at all (Figure 1).

**Table 3:** Level of significance, calculated using the χ^2^ test, of the differences in number of *atpD* sequences belonging to each group (A = Group A, B = Group B, C = Group C,) and *strA* sequences belonging to each group (1 = Group 1, 2 = Group 2, 3 = Group 3, O = others) between the main soil sites.

*atpD*			*strA*		
	CR	San		CR	San
AR	A = 0.0004 *	A = 1	AR	1 = 0.0037 *	1 = 0.0871
	B = 0.4443	B = 0.2771		2 = 0.4172	2 = 0.9430
	C = 0.0002 *	C = 0.2771		3 = 0.0808	3 = 0.0002 *
				O = 1	O = 0.0475 *
CR		A = 0.0022 *	CR		1<0.0001 *
		B = 0.8593			2 = 0.8501
		C = 0.0285 *			3<0.0001 *
					O = 0.0348 *

Significant probability values (p<0.05) are followed by an asterisk.

### Screening of soils for *strA* diversity

Two primer sets were used to detect *strA* (Table 1); the F2/R3 pair which was specific for the *strA* gene associated with the streptomycin biosynthetic cluster and the F/R pair, which was able to amplify from more diverse homologues to streptomycin 6-phosphotransferase genes such as hydroxyurea phosphotransferases [17].

While *atpD* was detected in TCDNA from soil, both sets of *strA* primers failed to produce any amplicons, demonstrating that *strA* was present below the detection threshold of the PCR used [36]. The *strA* gene was successfully amplified from seeded microcosms constructed from all soils studied. No amplicons were generated from extracted soil microcosm DNA using the strA_F2/R3 primers, whereas the more conserved strA_F/R primers amplified products of the correct size, as did the atpD_F/R primers.

### Phosphotransferase diversity detected using *strA* primers

**Figure 3:**
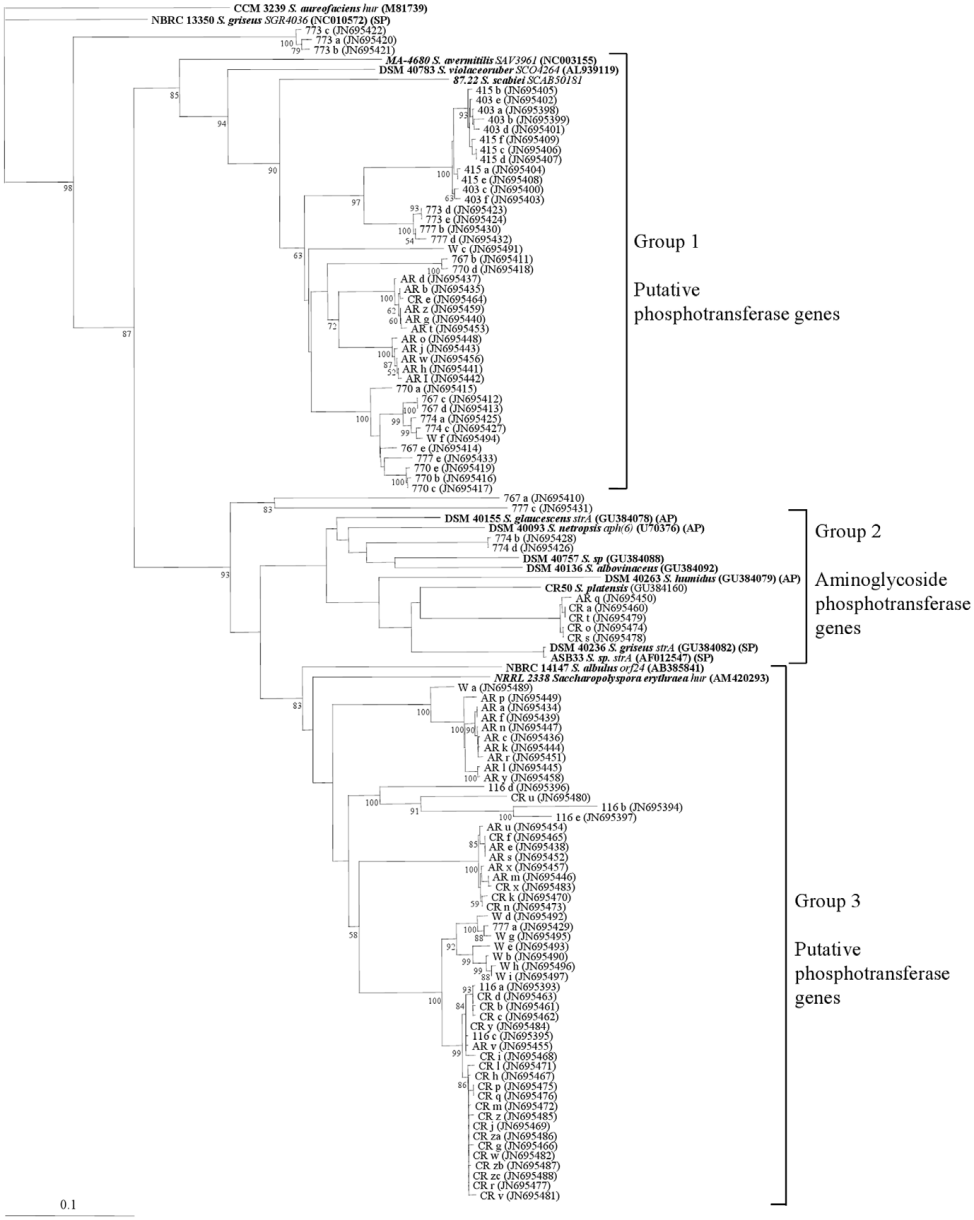
Phosphotransferase diversity in microcosm soil based on partial sequences derived from *strA* primers. Marker sequences (from GenBank) in bold. (SP) = Confirmed streptomycin producer, (AP) = Confirmed producer of other aminoglycoside [51], [52]. Accession numbers in parentheses. The tree was constructed using the neighbour-joining method; the numbers besides the branches indicate the percentage bootstrap value of 10000 replicates. The scale bar indicates 10% nucleotide dissimilarity.

All *strA* homologues that are part of an antibiotic gene cluster belonged to a single clade (Group 2) with a bootstrap value of 85 (Figure 3). Only 7 out of 105 sequenced amplicons (6.7%) belonged to this clade. Two Santomeras sequences formed a sister clade to a resistance-only *S. griseus strA* gene [17]. Four of the CR and an AR clone were in the same clade as the *strA* genes present in the streptomycin biosynthetic cluster of *S. griseus* DSM 40236 and *S. platensis* CR50, indicating that they may be associated with production. Of the remaining sequences, 40 (38%) formed a sister clade to *S. violaceoruber* DSM 40783 *SCO4264* (Group 1) and 51 (49%) formed a sister clade to a putative hydroxyurea phosphotransferase gene *hur* from *Saccharopolyspora erythraea* (Group 3). In addition there were two clades composed of 3 (2.9%) and 2 (1.9%) sequences that fell outside those three main groupings and were placed in the Outlier grouping.

**Figure 4:**
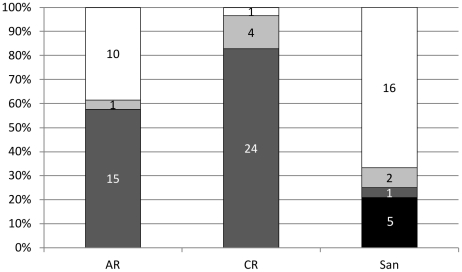
Distribution of the *strA* homologues isolated from the three main soil sites on the 3 main groups identified on the nucleotide phylogenetic tree (**Figure 3**). ‘San’ indicates all the Santomera strains (767, 770, 773, 774, 777). ‘Outliers’ refers to the two minor clades that do not form part of the 3 main groupings. Group 1 is in white, Group 2 in light grey, Group 3 in dark grey and the Outliers in black.

Sequences belonging to Group 1, 2 and 3 were isolated from both AR and CR, while the Santomeras soils additionally contained all the Outliers. There appeared to be large differences between sites (Figure 4), however when analyzed statistically there were only significant differences for Group 1 and 3 in 2 samples (Table 3). From the remaining sampled soils, 2 of the W sequences fell in Group 1 and 7 in Group 3, all 5 Skopelos sequences belonged to Group 3 and all 12 CB sequences to Group 1. The *strA* phylogenetic tree structure (Figure 3) demonstrates a correlation between site and diversity. The diversity followed a biogeographical distribution, and there was little separation between different soil sites from within an area. The one exception was the significant difference in number of AR and CR sequences in Group 1 despite both sites being in Dossenheim (Figure 4, Table 3). There were also significant differences in the proportions of *strA* homologue groups between different soil sites, demonstrating that the composition of the populations of these genes varies across different regions.

Two of the sequences (AR d and AR w) contained frameshift mutations and one (CR zb) contained a stop codon. The remaining sequences varied from the *S. griseus strA* and the *S. aureofaciens hur* but produced valid peptides. There was considerable variation in the peptide sequences which extended to the Mg^2+^ binding motif (Table 4a) (*S. griseus strA*: VLHWDLHYEN and in *S. aureofaciens hur*: LLHWDLHFGN) and the putative nucleotide-binding domain (Table 4b) (*S. griseus strA*: VGDPGFDLWP and in *S. aureofaciens hur*: AGDPGFELLP) [37]. This suggests that some of these sequences are pseudogenes and the products of others have targets that differ from those of StrA and Hur.

**Table 4:** Divergence of the (a) Mg^2+^ binding domain and (b) putative nucleotide-binding domain in environmental sequences compared to the *S. aureofaciens hur* peptide.

(a) Mg^2+^ binding domain	L	L	H	W	D	L	H	F	G	N
No. dissimilar	8	0	0	1	0	0	0	91	103	1
% dissimilar	7.8	0	0	1.0	0	0	0	88	100	1.0
(b) nucleotide-binding domain	A	G	D	P	G	F	E	L	L	P
No. dissimilar	18	1	1	0	0	0	60	0	17	1
% dissimilar	17	1	1.0	0	0	0	58	0	17	1.0

## Discussion

Use of the *atpD* housekeeper gene produced a tree with significant phylogenetic resolution. In addition, the majority of clades contained both sequences from TCDNA and characterized isolates, providing an insight into the *in situ* diversity of streptomycetes in soil. The *atpD* sequence data suggest that the most common streptomycin producer from isolation work, *S. griseus* DSM 40236 and its close relatives, is found at a low density in soil.However none of the cloned *atpD* genes matched or were related to *S. griseus* DSM 40236. The molecular detection limit for plant pathogenic streptomycetes, , in soil has been calculated to be 10^3^ gene targets per gram of soil for the *txtAB* gene [38], 1.5×10^3^ for the *nec1* gene [39] and 10^2^ for the 16 S gene [40], suggesting that *S. griseus* soil populations may be lower than these values even after enrichment with chitin and starch. Other studies also support this finding as TCDNA studies of 13 streptomycetes from soil crusts in the Colorado Plateau [41], 156 streptomycetes from prairie soil in the Cedar Creek Natural History Area [42], and an isolate study including 3204 isolates from Singapore rainforests [43] failed to detect *S. griseus*, while a TCDNA study of temperate forest soils from Italy found one *S. griseus* sequence among 22 streptomycetes [44], indicating that *S. griseus* is uncommon in soil.

Streptomycin producers are one of the most commonly isolated antibiotic producers from soil [33], however the current study failed to amplify any copies of *strA* that belong to the streptomycin gene cluster using either the *S. griseus* producer-specific primers or the more conserved *strA* primers. Therefore, despite its wide distribution, the streptomycin cluster is not common in soil streptomycetes at the sites examined. Streptomycin-resistant streptomycetes have been isolated from the Dossenheim soils using selective plates containing streptomycin [34]. The more diverse *strA* sequences were never found in isolates recovered from the same soils using streptomycin selection in a previous study [34]. The only gene, other than the *S. griseus* DSM 40236 *strA*, on the phosphotransferase tree whose function has been experimentally confirmed is the *hur* from *S. aureofasciens* which acts by phosphorylating the hydroxy group in the hydroxylamine moiety of hydroxyurea [45]. It has been speculated that its function in the natural environment is to inactivate an unknown aminoglycoside containing a domain resembling the hydroxylamine moiety [45]. The function of the other genes obtained from GenBank, e.g. *SCO4264* and *S. albulus* NBRC 14147 *orf24*, is unknown; these were listed as putative *hur* genes due to their sequence homology with *S. aureofasciens hur*. The detection of a small number of closely related homologues to *strA* indicates that these genes, which may form part of an aminoglycoside biosynthetic cluster or be independent, are more common than the *strA* belonging to the *S. griseus* streptomycin cluster. Nonetheless, they comprise only a minority of the genes encoding aminoglycoside/hydroxyurea antibiotic resistance kinases from the APH_6_hur superfamily. Thus potential streptomycin resistance genes not associated with a biosynthetic cluster are found in low numbers in the examined soils, which is to be expected if the streptomycin biosynthetic cluster is also not abundant. The sequences within the clade containing aminoglycoside producer resistance genes are likely to phosphorylate aminoglycosides, however the function of the remaining genes is less certain as they cluster with phosphotransferase genes whose substrate is unknown. Three studies used PCR and cloning to detect the presence of the 2-deoxy-scyllo-inosose gene in TCDNA from Japanese soils (54 clones), and Pacific Ocean (34 clones). None of these sequences were closely related to the *stsC* gene from the streptomycin cluster, instead forming clades with genes from other biosynthetic clusters such as kanamycin or gentamycin [11], [46], [47].

There have been few attempts to determine the prevalence of APH genes in the environment which can both provide information on the frequency of resistant strains as well as assist in assessing the frequency of aminoglycoside producers. In an agricultural field site from Costa Rica, two from a set of 69 actinobacterial isolates (2.9%) possessed an *strA* homologue that was more closely related to the *S. glaucescens strA*, while none of the 48 clones from a coastal salt marsh from the USA were closely related to *strA* [37]. DNA extracted from three Swedish wastewater-associated environments were screened for the presence of the aminoglycoside resistance genes *aac(6′)-Ie* and *aph(2″)*. The genes were detected at all the sites, though the soil had very low levels of the genes [48]. A BAC library containing 5.6 Gb of DNA extracted from soil contained 9 clones, of which 6 were AAC(6′), and none were APH genes [49]. The fungus/bacterium ratio, based on abundance of rRNA estimated to range from 0.2 for desert and 0.6 for prairie soil to 2 to 4 for forest soil (Assessment of Soil Microbial Community Structure by Use of Taxon-Specific Quantitative PCR Assays, Soil Microbial Community Responses to Multiple Experimental Climate Change Drivers), Assuming that an average bacterial chromosome is 4.5 Mb [50] and a fungus/bacterium ratio of 4 for the oak savanna examined in that study, it can be estimated that only 0.4% of soil bacteria have resistance to aminoglycosides due to enzymatic modification of the antibiotic. These findings agree with the results of this study and indicate that APH(6′) resistance genes are not prevalent in soil.

The streptomycin-treated and untreated sites demonstrated significant differences in the composition of both the *strA* gene and the *atpD* gene. The significantly lower number of *strA* sequences putatively associated with production suggests that other phosphotransferase genes are selected for in the presence of streptomycin, resulting in a relatively smaller population of streptomycin producers. In addition, the significant difference in the composition of *atpD* and *strA* sequences, despite the fact both sites were in the same region, suggests that the application of plantamycin may have caused alteration in the soil streptomycete populations, as there were no significant differences between the different Santomera sampling sites.
